# Ubiquitination Regulates the Morphogenesis and Function of Sperm Organelles

**DOI:** 10.3390/cells2040732

**Published:** 2013-12-05

**Authors:** Nobuhiro Nakamura

**Affiliations:** Department of Biological Sciences, Tokyo Institute of Technology, 4259-B13 Nagatsuta-cho, Midori-ku, Yokohama 226-8501, Japan; E-Mail: nnakamur@bio.titech.ac.jp; Tel.: +81-45-924-5726; Fax: +81-45-924-5824.

**Keywords:** acrosome, deubiquitination, histone, membrane trafficking, mitochondria, mitophagy, spermatogenesis, spermiogenesis, ubiquitination

## Abstract

It is now understood that protein ubiquitination has diverse cellular functions in eukaryotes. The molecular mechanism and physiological significance of ubiquitin-mediated processes have been extensively studied in yeast, *Drosophila* and mammalian somatic cells. Moreover, an increasing number of studies have emphasized the importance of ubiquitination in spermatogenesis and fertilization. The dysfunction of various ubiquitin systems results in impaired sperm development with abnormal organelle morphology and function, which in turn is highly associated with male infertility. This review will focus on the emerging roles of ubiquitination in biogenesis, function and stability of sperm organelles in mammals.

## 1. Introduction

Protein ubiquitination has long been recognized as one of the key determinants of protein stability and activity. This post-translational modification plays a critical role in a variety of cell functions, such as cell proliferation, cell differentiation, signal transduction, protein trafficking, immune response and apoptosis. The conjugation of ubiquitin to target proteins (or itself) is evolutionally conserved and has the following characteristics. It is mediated through the sequential activities of three ubiquitinating enzymes: ubiquitin-activating enzyme (E1), ubiquitin-conjugating enzyme (E2) and ubiquitin ligase (E3) [1]. To date, broad range of proteins have been reported to undergo ubiquitination, including receptors, transcription factors, ion channels, signaling molecules, cytoskeletal components and unnecessary/damaged proteins. E3 enzymes are key factors that determine substrate specificity, and for this reason the human genome encodes more than 600 E3s [2]. In addition to the high number of E3s, the different modes of ubiquitin conjugation reflect the functional diversity of protein ubiquitination. Proteins can be modified by addition of single ubiquitin molecule (monoubiquitination) or a polymer of ubiquitin (polyubiquitination). Ubiquitin contains seven lysine residues (K6, K11, K27, K29, K33, K48 and K63) that can be conjugated to another ubiquitin molecule, thereby forming at least seven different polyubiquitin linkages [3]. A K48-linked polyubiquitin chain generally serves as a signal for protein degradation by the proteasome. Monoubiquitination and K63-linked ubiquitination have various non-proteasomal functions, such as endocytosis, protein trafficking and DNA repair. Other ubiquitin linkages are comparatively minor, but they appear to function in proteasomal degradation and DNA repair [4]. Ubiquitination is counteracted by deubiquitinating enzymes that remove ubiquitin from protein substrates [5]. The molecular mechanism and physiological significance of ubiquitin-mediated processes have been extensively studied in yeast, *Drosophila* and mammals, but have been much less investigated in germ cells. However, over the years, an increasing number of studies have emphasized the importance of the ubiquitin system in male gametogenesis (spermatogenesis) and fertilization. Sperm have unique membranous organelles specialized for sperm motility and penetration, that is, a condensed nucleus, an acrosome and helically arranged mitochondria. Defects in organization and/or integrity of these organelles are closely associated with impaired sperm function and male infertility. In somatic cells, protein ubiquitination plays a central role in the regulation of the morphology and function of membranous organelles, for example, protein quality control in the endoplasmic reticulum (ER), protein sorting in endosomes and mitochondrial dynamics [6,7,8]. Likewise, it could be important for the maintenance of the integrity of sperm organelles. This review will focus on recent advances in the state of knowledge concerning the role of protein ubiquitination in biogenesis and the function and stability of sperm membranous organelles.

## 2. Ubiquitinating and Deubiquitinating Enzymes during Spermatogenesis

**Figure 1:**
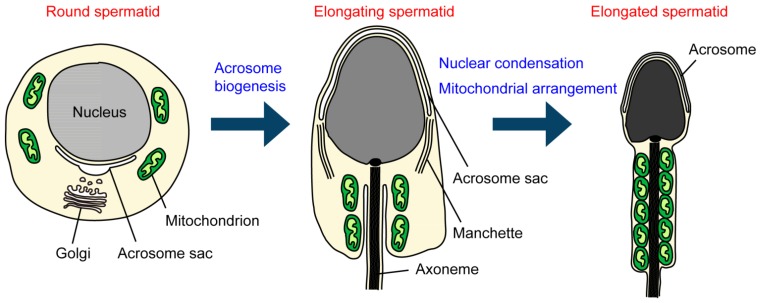
Biogenesis and morphological changes in intracellular membranous organelles during spermiogenesis. In round spermatids, the acrosome sac is formed and enlarged by the continuous fusion of Golgi-derived vesicles. In elongating spermatids, the acrosome sac then flattens and covers the anterior half of the nucleus. In the nucleus, histone-to-protamine replacement occurs, which allows the nucleus to become condensed with the aid of the manchette structure. In elongated spermatids, mitochondria are aligned along the anterior part of the flagellum (the midpiece) and tightly packed to form the helically arranged mitochondrial sheath.

Spermatogenesis is a complex and dynamic process by which the metamorphosis of male germ cells into mature spermatozoa takes place. During mammalian spermatogenesis, spermatogonial stem cells proliferate and differentiate into spermatocytes by mitotic division. Subsequently, diploid spermatocytes undergo meiosis and differentiate into haploid round spermatids. Round spermatids transform into elongated spermatids through a unique differentiation process called spermiogenesis, and then eventually develop into mature spermatozoa. Spermiogenesis includes nuclear shaping, acrosome biogenesis, flagellum formation, mitochondrial rearrangement and cytoplasmic trimming (Figure 1) [9]. Several testis transcriptome studies have shown that the complex process of spermatogenesis is regulated by a highly integrated mechanism that involves changes in gene expression in a developmental stage-dependent manner [10,11,12]. An increasing number of gene products have been found to have specialized biological functions required for the different phases of spermatogenesis. Indeed, a recent proteomics study identified more than 4,600 human sperm proteins, of which approximately 220 are sperm-specific [13]. Since ubiquitin was first isolated from the testes of trout and mammals [14,15], more than 30 ubiquitinating enzymes have been identified as important regulators of spermatogenesis [16]. It is estimated that approximately 70 E3 ubiquitin ligases are expressed during spermatogenesis in mice [17], suggesting that the ubiquitin system has diverse functions. Further investigation is needed to address the physiological roles of the as yet uncharacterized sperm E3s. Mammals contain ~90 deubiquitinating enzymes, many of which are associated with pathogenesis [18]. At present, little is known about the expression and physiological roles of deubiquitinating enzymes in male gamete cells. Several deubiquitinating enzymes have been identified as important regulators of spermatogenesis and both their mutations and targeted disruption reportedly cause severe abnormalities in sperm development and fertility [19,20,21,22,23,24,25]. A recent study reported the expression of 205 genes of the ubiquitin system in gonocytes and spermatogonia [26]. Among them, 91 genes with a relatively high expression included not only E3s but also deubiquitinating enzymes, such as ubiquitin-specific protease (USP)2 and USP19, suggesting that deubiquitination has an important role in gonocyte differentiation [26].

## 3. Roles of Ubiquitination in Sperm Organelles

### 3.1. Histone Ubiquitination for the Purpose of Nuclear Condensation

The head of a sperm contains a highly condensed nucleus with densely packed genomic DNA, which facilitates both its protection from nucleases and mutagens and its transportation to an oocyte [27,28]. Nuclear condensation is believed to be achieved by coordinated spermatogenesis-specific processes including chromatin condensation, acrosome formation and the remodeling of cytoskeletal structures [29]. Chromatin condensation is mediated by the removal of the histone from the nucleosome, where histones are initially replaced by transition proteins and subsequently by protamines, small arginine-rich proteins. Owing to the large number of positively charged residues, protamines bind to and neutralize negatively charged DNA, resulting in tighter chromatin packaging in the sperm nucleus [30]. Histone ubiquitination has been shown to play an important role in chromatin condensation during spermatogensis. Histone H2A is ubiquitinated in pachytene spermatocytes during the first meiosis and in elongating spermatids just prior to histone-to-protamine transition [31]. In yeast, the Rad6 (Ubc2) E2 enzyme catalyzes the monoubiquitination of histone H2B in conjunction with the Bre1 E3 enzyme [32,33,34,35,36]. Rad6/Bre1-mediated monoubiquitination of H2B triggers the subsequent methylation of histone H3 [36,37,38,39], leading to transcriptional activation and elongation [40,41,42,43]. This function is evolutionally conserved throughout the higher eukaryotes [44,45]. Although Rad6 catalyzes the ubiquitination of both H2A and H2B *in vitro* [32,33], H2A ubiquitination is not detected in yeast. Numerous studies have demonstrated that H2A ubiquitination has a role in the transcriptional silencing that takes place in higher eukaryotic cells [44,45]. HR6B (UBE2B), a mammalian homolog of yeast Rad6, is highly expressed in elongating spermatids and is localized to the euchromatin area [46]. In mice, disruption of HR6B results in male sterility due to impaired spermatogenesis, with extensive apoptosis of spermiogenic cells and abnormal sperm head morphology [47]. Incomplete histone displacement occurs in the nucleus of *HR6B*-deficient sperm [47]. These observations suggest that ubiquitination by HR6B is essential for histone eviction and subsequent nuclear condensation in elongating spermatids. In post-meiotic spermatids, histone ubiquitination is likely to facilitate the relaxation of chromatin structures, which increases the accessibility for transient proteins/protamines as well as transcriptional regulators. It is known that histones are heavily acetylated prior to their replacement and then disappear in elongating spermatids [48]. Surprisingly, Qian *et al.* [49] have recently shown that acetylation and not ubiquitination serves as the signal for proteasomal degradation during spermatogenesis. The proteasome activator PA200 binds to the end of the 20S proteasome [50] and recognizes acetylated histones for proteasomal degradation [49]. In mice, deletion of PA200 causes reduced male fertility due to impaired spermatogenesis [51]. In addition, an increased level of certain histones, including acetylated histone H4, remain in the elongating spermatids of PA200 deficient mice [49]. Lu *et al.* [52] have provided evidence for a close link between H2A ubiquitination and H4 acetylation. They have shown that the RNF8 E3 enzyme catalyzes H2A ubiquitination and simultaneously promotes H4 acetylation through binding to the H4K16 acetyltransferase MOF (males absent of the first) [52].

Apart from chromatin remodeling in post-meiotic spermatids, histone ubiquitination has a critical role in transcriptional silencing during meiosis. During the pachytene stage of the meiotic prophase, HR6B and H2A ubiquitination are enriched in the XY body, where the X and Y chromosomes partially associate to form a synapsis in their short pseudoautosomal regions [31,53]. Upon forming the XY body, the sex chromosomes are silenced through a process called meiotic sex chromosome inactivation (MSCI) [54]. Gene disruption of the mouse UBR2 E3 ubiquitin ligase results in male infertility and incomplete spermatogenesis, a condition in which spermatocytes are arrested before the pachytene stage and die via apoptosis [55]. UBR2 has also been shown to interact with HR6B and mediate MSCI by ubiquitinating H2A [56]. However, recent studies have suggested that H2A ubiquitination is not required for MSCI [52,57]. In addition, HR6B inactivation affects the methylation and phosphorylation of histones but not H2A ubiquitination, suggesting it has a role in the maintenance of postmeiotic silencing [58]. On the other hand, in *HR6B*-deficient pachytene spermatocytes, the synaptonemal complexes (tripartite protein structures that form between homologous chromosomes during meisosis) become longer and the number of meiotic recombination sites increases, suggesting that HR6B may have an inhibitory role in meiotic recombination [59]. RAD18, RNF20 and RNF40 have been identified as putative additional partner E3s for HR6B. RAD18 is enriched in the XY body in mouse pachytene spermatocytes and has thus been proposed to be involved in meiotic transcriptional silencing [60]. RNF20 and RNF40 are mammalian homologs of yeast Bre1, but their expression and function during spermatogenesis are unknown [61]. It has been shown that RNF8 catalyzes H2A ubiquitination on the XY body and its activity is required for the initial step of nucleosome removal [52]. LASU1 (also named MULE-1, ARF-BP1 or HUWE1) is another candidate E3 for H2A ubiquitination. LASU1 is expressed in the nucleus from the stage of spermatogonia to pachytene spermatocytes and appears to ubiquitinate H2A in a manner dependent on the E2 activity of the testis-specific isoform of UBC4 [62,63].

In light of these results, it is clear that histone ubiquitination is indispensable for chromatin remodeling during mammalian spermatogenesis. To understand the relationship between ubiquitination and nuclear condensation, further studies will be required to clarify the precise mechanisms underlying meiotic transcriptional silencing and histone eviction.

### 3.2. Acrosome Formation and Membrane Trafficking

#### 3.2.1. TMF/ARA160—A Mediator of Fusion of Golgi-Derived Vesicles

**Figure 2:**
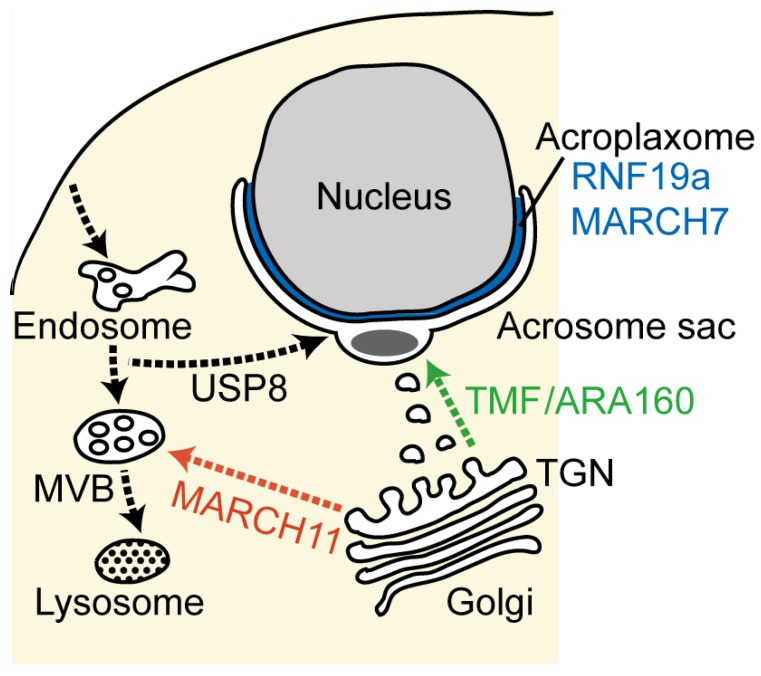
Schematic diagram of the transport pathways during acrosome formation. The major membranous organelles and intracellular transport pathways are indicated, with the anterograde biosynthetic pathway in green, the endocytic retrograde pathway in black and the *trans*-Golgi network (TGN)-to-multivesicular body (MVB) pathway in red. The reported ubiquitinating and deubiquitinating enzymes involved in these transport pathways are indicated.

The acrosome is a large, flattened vesicle overlaying the anterior portion of the sperm nucleus. It contains hydrolytic enzymes essential for penetration into the ovule. Acrosome formation begins at the early stages of spermiogenesis, with proacrosomal vesicles (also called proacrosomal granules) derived from the Golgi apparatus. Numerous newly synthesized acrosomal enzymes, such as acrosin, are sorted and packed from the *trans*-Golgi network (TGN) into the proacrosomal vesicles. The single acrosomal sac is formed by attachment of the proacrosomal vesicle to the nuclear envelope and then enlarges as more Golgi-derived proacrosomal vesicles fuse (Figure 2). The acrosome gradually becomes flattened and ultimately spreads to cover the anterior half of the nucleus. The assembly and fusion of the proacrosomal vesicles are most likely controlled by membrane trafficking machinery, including Rab and SNARE (soluble *N*-ethylmaleimide sensitive factor attachment protein receptor) proteins, which are associated with the Golgi apparatus and acrosome [64,65,66]. Perturbation of the membrane flow to the developing acrosome results in disruption of the acrosome, and infertile and round-headed spermatozoa (globozoospermia) [67,68,69,70,71]. Lack of acrosome formation has also been observed in mice with a disrupted TMF/ARA160 (TATA element modulatory factor/androgen receptor co-activator 160 kDa) gene, which encodes a Golgi-localized coiled-coil protein [72]. In somatic cells, TMF/ARA160 participates in the tethering and targeting of transporting vesicles at the Golgi by forming a complex with Rab6 and the conserved oligomeric Golgi (COG) complex [73,74,75]. In TMF-deficient spermatids, Golgi-derived proacrosomal vesicles neither fuse with each other nor attach to the nuclear envelope, either or both of which might be due to lack of the TMF-Rab6-COG complex [72]. It is noteworthy that TMF exerts an E3 ubiquitin ligase activity upon association with an E3 ubiquitin ligase complex in stressed somatic cells [76,77]. Although no substrate proteins have yet been found for the E3 activity in spermatogenic cells, TMF-mediated protein ubiquitination may be involved in acrosome biogenesis.

#### 3.2.2. MARCH11—Regulation of Golgi-to-Endosome Transport

A subset of newly synthesized proteins is sorted out from the Golgi-to-acrosome pathway in order to be delivered to the endosomal system. In fact, some late endome/lysosome proteins, such as mannose 6-phosphate receptors and lysosome-associated membrane proteins (LAMPs), are segregated from the acrosome. In yeast and somatic cells, monoubiquitin or K63-linked ubiquitin tags serve as sorting signals for the delivery of transmembrane proteins to the multivesicular bodies (MVBs), which are parts of the endosome system. In the MVB pathway, ubiquitinated cargo proteins are incorporated into the intraluminal vesicles of the MVBs by sequential sorting machinery called “endosomal sorting complexes required for transport” (ESCRT), and are subsequently transported to the lysosomes [8,78]. In spermiogenesis, the presence of the MVBs is evident, but little is known about the sorting mechanism or the cargo proteins in the MVB pathway. Given the fact that ubiquitinated proteins are present in the TGN, acrosome and MVBs [79], protein ubiquitination is likely to contribute to protein sorting in both the acrosome and MBV pathways. Recent studies have shown that TGN-to-MVB protein transport is regulated by membrane-associated RING-CH (MARCH) 11, a member of the transmembrane E3 ubiquitin ligase family [80,81]. Generally, MARCH proteins control cell-surface expression of receptor molecules by mediating ubiquitination and subsequent lysosomal degradation [6,82]. In early round spermatids, MARCH11 is localized to the TGN and MVBs, and ubiquitinates spermatogenesis-associated multicopy transmembrane (SAMT) proteins which are delivered to the late endosomal/lysosomal compartments [80,81]. Cytochemical studies have demonstrated that fucose-containing proteins are transported from the TGN to the MVBs during acrosome formation [83,84]. MARCH11 has been shown to associate with fucose-containing proteins, including the ubiquitinated forms [80], suggesting that it may control the sorting and targeting of certain glycoproteins destined for the MVB pathway.

In our previous reverse-transcriptase PCR experiments, gene expression of other transmembrane MARCH E3s (*i.e.*, MARCH2, 3, 5, 6, 8 and 9) were detected in the rat testis, but there is no information on their expression, subcellular localization and/or functional roles in male germ cells [80]. It is at least known that MARCH7 and MARCH10, which are non-transmembrane proteins, are specifically expressed in elongating spermatids in mid-to-late spermiogenesis and are associated with the acroplaxome (see Section 3.2.4) and flagella, respectively, suggesting their possible involvement in head shaping and flagellar formation [85,86]. Thus, it may be speculated that MARCH E3s play diverse roles in spermatogenesis.

#### 3.2.3. USP8/UBPy—The Retrograde Pathway for Acrosome Formation

It is believed that vesicular transport from the Golgi apparatus is indispensable for the acrosome formation, but recent reports have pointed out the additional involvement of other trafficking pathways from the plasma membrane and endosomes [87,88,89,90]. In particular, the deubiquitinating enzyme USP8 (also named UBPy) has been shown to be localized to the early endosomes and the acrosome during acrosome biogenesis [90,91]. In somatic cells, USP8 interacts with the ESCRT-0 machinery [*i.e.*, STAM (signal transducing adaptor molecule) and HGS (hepatocyte growth factor-regulated tyrosine kinase substrate)] and its deubiquitinating activity regulates the endosomal sorting of ubiquitinated cargo between the recycling pathway and the lysosomal degradation pathway [92]. Spermatid USP8 is also associated with ESCRT-0 through binding to STAM-2, and they are co-localized on the early endosome in early round spermatids and later accumulate in the acrosome [90]. Thus, USP8 is likely to be involved in acrosome formation by controlling the sorting and ubiquitination status of cargo proteins in the endosome-to-acrosome trafficking pathway and/or at the developing acrosome. Membrane flow to the acrosome depends on vacuolar protein sorting (Vps) 54, a component of the Golgi associated retrograde protein (GARP) complex that is required for retrograde traffic from endosomes to the TGN in yeast and somatic cells [93]. The GARP complex tethers retrograde transporting vesicles with the acceptor membrane by interacting with the membrane trafficking machinery, including Rab and SNARE. A misssense mutation (L967Q) in the mouse gene *Vps54* is the cause of the wobbler mouse phenotype that exhibits motor neuron degeneration and impaired spermiogenesis along with disorganization of the acrosome [94,95,96,97]. In wild type mice, Vps54 is colocalized with USP8 and ESCRT-0 in vesicular structures in the cytoplasm of round spermatids and in the acrosome of elongating spermatids [90]. On the other hand, the accumulation of USP8 and Vps54 does not occur in wobbler mouse spermatids [97]. It has long been accepted that acrosome biogenesis is accomplished by the supply of membrane and protein components via the biosynthetic pathway directly to the acrosome, but these studies suggest a contribution of the retrograde pathway from the plasma membrane and endosomes, possibly including the MVBs.

#### 3.2.4. RNF19a and MARCH7—Acroplaxome-Localized E3s

As the acrosome grows, it flattens and spreads over the anterior half of the elongating nucleus. A tight association of the acrosomal membrane with the nuclear envelope is mediated by the acroplaxome (also known as the subacrosomal layer), an F-actin and keratin 5 containing cytoskeletal plate (Figure 2). Since the acroplaxome contains the actin-based motor protein myosin Va and the membrane trafficking regulators Rab27a/b, it is thought to provide a transport pathway for the proacrosomal vesicles [69,98]. The elongating sperm head possesses another cytoskeletal structure, the manchette. The manchette is a transient microtubule-based skirt-like structure attached caudally to the acroplaxome. Given the presence of the kinesin motor proteins (KIFC1, KIF17b and KIF3A) [99,100,101], as well as cytoplasmic dynein [102,103], the manchette is believed to not only mediate cargo transport to the acrosome, but also provide driving force for nuclear elongation and acrosome extension. Thus, the acroplaxome and manchette complex is likely to be indispensable for acrosome formation and sperm head shaping. Recent studies have suggested that the ubiquitin–proteasome system is involved in the organization and function of the acroplaxome. Rivkin *et al.* [104] have identified RNF19a E3 ubiquitin ligase (also named Dorfin) as a component of the acrosome and acroplaxome. RNF19a is present on the Golgi-derived proacrosomal vesicles and later on the outer acrosomal membrane and the acroplaxome in the spermatids and spermatozoon of rats. In these structures, RNF19a forms a complex with Psmc3 (also named TBP-1), a subunit of the 26S proteasome [104]. In addition, ubiquitination, including K48-linked ubiquitination, is detected in the acrosome/acroplaxome regions [79,86,104]. RNF19a may thus mediate the quality control of abnormal or unassembled acrosome/acroplaxome proteins by targeting them for proteasomal degradation. In addition to the acrosome/acroplaxome localization, RNF19a is also present in the manchette, centriole and flagellum, suggesting it may also play a role in intraflagellar transport or sperm tail formation [104]. Likewise, MARCH7 E3 ubiquitin ligase has been shown to be localized to the acroplaxome and flagellum in the elongating spermatids and spermatozoon of rats [86]. MARCH7 catalyzes the formation of K48-linked ubiquitin chains, suggesting its possible role in protein degradation [86]. However, the identification of substrate proteins of both RNF19a and MARCH7 has not yet been reported, and is needed to elucidate their functions in the mechanism underlying acrosome and flagellar formation.

### 3.3. Maternal Inheritance of Mitochondrial DNA

#### 3.3.1. Ubiquitination of Sperm Mitochondria

Mitochondria are essential organelles that generate most of the cell’s supply of energy (ATP) obtained by oxidative phosphorylation. They also play other important cellular roles, including lipid and amino acid metabolism, calcium buffering, energy transmission, signal transduction and apoptosis. Mitochondria have multiple copies of their own genomic DNA (mitochondrial DNA; mtDNA) that encodes 13 essential components of the oxidative phosphorylation system, 22 transfer RNAs and 2 ribosomal RNAs; the majority of mitochondrial proteins are encoded by nuclear genomic DNA. Mitochondria often generate reactive oxidative species (ROS) as a toxic byproduct of oxidative phosphorylation. ROS damage mitochondrial DNA, proteins and lipids, leading to mitochondrial dysfunction. Damaged mitochondria release calcium ions and pro-apoptotic molecules, such as cytochrome *c*, into the cytosol, which triggers apoptosis. Thus, an accumulation of mtDNA mutations is associated with aging and neurodegenerative diseases [105].

In mammals, sperm mitochondria are assembled into tightly packed spirals (the mitochondrial sheath) around the midpiece of the flagellum. This unique structure enables a quick and efficient energy supply for sperm motility. However, it should be noted that there is a growing body of evidence that sperm rely on glycolysis rather than oxidative phospholyration for their energy production [106,107,108]. Since sperm need a great deal of energy for both their survival and rapid movement, sperm mtDNA is vulnerable to an accumulation of damaging ROS. Mitochondrial DNA is therefore transmitted maternally to offspring although paternal mtDNA does enter the oocyte at fertilization [109,110,111]. The mechanism for maternal mtDNA inheritance appears to involve the low copy number of sperm mtDNA (10–1,000 copies in a sperm compared to 100,000–1,000,000 copies in an oocyte) [112,113,114] and also the degradation of paternal mtDNA in fertilized eggs. Sutovsky *et al.* have suggested that paternal mtDNA is selectively eliminated by ubiqutination and subsequent proteasomal or lysosomal degradation of sperm mitochondria [115,116]. Sperm-derived mitochondria are degraded during the very early stages of embryogenesis (4- to 8-cell stages) and this degradation is blocked by the microinjection of anti-ubiquitin antibody or by treatment of fertilized eggs with ammonium chloride, a lysosome inhibitor [116]. Ubiquitination of mitochondria already occurs in spermatocytes [116]. Recent studies have detected the formation of K48-linked ubiquitin chains in the midpiece region of rat elongated spermatids and mouse spermatozoa [86,117], supporting the idea that paternal mitochondria are recognized by the oocyte proteasome. Sutovsky *et al.* [116] have identified prohibitin, a membrane protein of sperm mitochondria, as a substrate of sperm mitochondrial ubiquitination. However, additional substrate proteins and ubiquitinating enzymes responsible for sperm mitochondrial ubiquitination have yet to be reported. MARCH5 appears to be a candidate E3 ubiquitin ligase, because it is a bona fide mitochondrial E3 that targets several mitochondrial proteins for ubiquitination in somatic cells [118,119]. Of course, we cannot rule out the possible involvement of other non-mitochondrial E3s, such as Parkin (see the next section). Besides the molecular identity of the ubiquitinating enzymes, many questions still remain unanswered, for example, when sperm mitochondrial ubiquitination occurs, why mitochondria are not degraded in sperm and what mechanism ensures the recognition and degradation of paternal mitochondria in fertilized eggs.

#### 3.3.2. Does “Mitophagy” Eliminate Paternal Mitochondria?

Recently, selective autophagic degradation of mitochondria (mitophagy) has received significant attention as a mechanism to selectively eliminate damaged mitochondria in yeast, *Drosophila* and mammalian somatic cells. One pathway of mitophagy is mediated through the ubiquitination of mitochondria. Loss of the mitochondrial membrane potential in damaged mitochondria leads to accumulation of PTEN-induced putative kinase 1 (PINK1) on the outer mitochondrial membrane, which promotes the recruitment of Parkin E3 ubiquitin ligase. Parkin then ubiquitinates mitochondrial proteins at the outer mitochondrial membrane, such as voltage-dependent anion channel (VDAC) and mitofusins, thereby leading to the assembly of the autophagic machinery for mitophagy (p62 and LC3) [120,121]. The engulfed mitochondria are degraded upon fusion of the autophagosome with the lysosome. Given the activities of ubiquitin- and lysosome-dependent mitochondrial degradation [116], one can envision a mechanism that removes sperm-derived mitochondria via mitophagy after fertilization. Recent studies in *Caenorhabditis elegans* reported that sperm mitochondria are degraded by mitophagy upon fertilization [122,123]. However, ubiquitination occurs on nematode-specific sperm-derived membranous organelles (MOs) but not on mitochondria, which triggers autophagosome formation. Since mitochondria are located in close proximity to MOs, they are subjected to mitophagic degradation along with them [122,123]. In the case of mammals, there is no clear consensus whether sperm-derived mitochondria are eliminated through mitophagy. In mice, autophagy is upregulated after fertilization and is essential for early embryogenesis [124]. In addition, fertilization induces K63-linked ubiquitination, which is known to be catalyzed by Parkin [125,126], and then the recruitment of p62 and LC3 to the midpiece of mouse spermatozoa [123]. However, a recent study by Luo *et al.* [117] has demonstrated that even though mouse sperm mitochondria are ubiquitinated and recruit p62 and LC3 immediately after fertilization, they are not engulfed by autophagosomes and eventually are degraded in lysosomes. Furthermore, the study also showed that paternal mtDNA, but not mitochondria, is eliminated before fertilization, and that degradation of paternal mitochondria is not essential for paternal mtDNA elimination [117]. It is thus possible that mammals may have acquired different mechanisms to strictly ensure maternal mtDNA inheritance. Yet it remains unknown whether these mechanisms are conserved across species. Further studies should be performed to clarify the correlation of mitochondrial ubiquitination with the degradation of sperm mitochondria and mtDNA, and to determine if it plays other roles in spermatogenesis, sperm function, fertilization and embryogenesis.

## 4. Conclusions

Based on published reports, it is clear that the ubiquitin system has a central role in the key events in spermatogenesis and fertilization. In somatic cells, an extensive number of studies have demonstrated that protein ubiquitination plays a key role in controlling protein stability, localization and activity. Although the role of protein ubiquitination is less clear in male germ cells, ubiquitinating and deubiquitinating enzymes have been shown to regulate the biogenesis, maintenance, stability and function of membrenous organelles, including acrosome formation, nuclear condensation, intracellular membrane trafficking and paternal mitochondrial elimination. Besides the ubiquitin system described here, a number of sperm ubiquitinating and deubiquitinating enzymes have been identified and shown to be involved in other biological events required for spermatogenesis (e.g., flagellar formation), sperm quality control and fertilization [16,127,128,129,130]. Researchers in the field of reproductive biology often face difficulties with the culturing and transfection of spermatogenic cells and sperm. Therefore, direct evidence for the involvement of the ubiquitin system is thus mostly provided by the phenotypes of mutant or knockout mice. Analysis of sperm genetics and organelle function requires specific techniques that are able to isolate male germ cells at a specific step and then specific organelles from them as well. However, future biochemical characterization and localization studies of (de)ubiquitinating enzymes and their substrates will shed light on understanding the mechanism and impact of ubiquitin-mediated regulation of sperm organelles. The data by DNA microarray and proteomics will help to elucidate the identities of the components of sperm organelles. Moreover, given the fact that different patterns of ubiquitin conjugation confer different biological functions, it will be necessary to elucidate the type of protein ubiquitination (e.g., poly-, mono-, K48-linked or K63-linked ubiquitination) is used for each event in spermatogenesis and fertilization. Future increases in our knowledge of the functions of sperm protein ubiquitination will provide insight into the pathogenesis of male infertility, and thus ultimately, effective treatment.

## References

[1] Glickman M.H., Ciechanover A. (2002). The ubiquitin-proteasome proteolytic pathway: Destruction for the sake of construction. Physiol. Rev..

[2] Li W., Bengtson M.H., Ulbrich A., Matsuda A., Reddy V.A., Orth A., Chanda S.K., Batalov S., Joazeiro C.A. (2008). Genome-wide and functional annotation of human E3 ubiquitin ligases identifies MULAN, a mitochondrial E3 that regulates the organelle's dynamics and signaling. PLoS One.

[3] Ikeda F., Dikic I. (2008). Atypical ubiquitin chains: New molecular signals. ‘Protein Modifications: Beyond the Usual Suspects’ review series. EMBO Rep..

[4] Kulathu Y., Komander D. (2012). Atypical ubiquitylation—the unexplored world of polyubiquitin beyond Lys48 and Lys63 linkages. Nat. Rev. Mol. Cell Biol..

[5] Reyes-Turcu F.E., Ventii K.H., Wilkinson K.D. (2009). Regulation and cellular roles of ubiquitin-specific deubiquitinating enzymes. Annu. Rev. Biochem..

[6] Nakamura N. (2011). The role of the transmembrane RING finger proteins in cellular and organelle function. Membranes.

[7] Vembar S.S., Brodsky J.L. (2008). One step at a time: Endoplasmic reticulum-associated degradation. Nat. Rev. Mol. Cell Biol..

[8] Shields S.B., Piper R.C. (2011). How ubiquitin functions with ESCRTs. Traffic.

[9] Clermont Y., Oko R., Hermo L. (1993). Cell Biology of Mammalian Spermatogenesis. Desjarjins C and Ewing LL (ed) Cell. and Molecular Biology of the Testis.

[10] Schultz N., Hamra F.K., Garbers D.L. (2003). A multitude of genes expressed solely in meiotic or postmeiotic spermatogenic cells offers a myriad of contraceptive targets. Proc. Natl. Acad. Sci. USA.

[11] Shima J.E., McLean D.J., McCarrey J.R., Griswold M.D. (2004). The murine testicular transcriptome: Characterizing gene expression in the testis during the progression of spermatogenesis. Biol. Reprod..

[12] Pang A.L., Johnson W., Ravindranath N., Dym M., Rennert O.M., Chan W.Y. (2006). Expression profiling of purified male germ cells: Stage-specific expression patterns related to meiosis and postmeiotic development. Physiol. Genomics.

[13] Wang G., Guo Y., Zhou T., Shi X., Yu J., Yang Y., Wu Y., Wang J., Liu M., Chen X. (2013). In-depth proteomic analysis of the human sperm reveals complex protein compositions. J. Proteomics.

[14] Roth G., Himstedt W. (1978). Response characteristics of neurons in the tectum opticum of Salamandra. Naturwissenschaften.

[15] Loir M., Caraty A., Lanneau M., Menezo Y., Muh J.P., Sautiere P. (1984). Purification and characterization of ubiquitin from mammalian testis. FEBS Lett..

[16] Hou C.C., Yang W.X. (2013). New insights to the ubiquitin-proteasome pathway (UPP) mechanism during spermatogenesis. Mol. Biol. Rep..

[17] Hou X., Zhang W., Xiao Z., Gan H., Lin X., Liao S., Han C. (2012). Mining and characterization of ubiquitin E3 ligases expressed in the mouse testis. BMC Genomics.

[18] Clague M.J., Barsukov I., Coulson J.M., Liu H., Rigden D.J., Urbe S. (2013). Deubiquitylases from genes to organism. Physiol. Rev..

[19] Sun C., Skaletsky H., Birren B., Devon K., Tang Z., Silber S., Oates R., Page D.C. (1999). An azoospermic man with a de novo point mutation in the Y-chromosomal gene USP9Y. Nat. Genet..

[20] Foresta C., Ferlin A., Moro E. (2000). Deletion and expression analysis of AZFa genes on the human Y chromosome revealed a major role for DBY in male infertility. Hum. Mol. Genet..

[21] Stouffs K., Lissens W., Tournaye H., van Steirteghem A., Liebaers I. (2005). Possible role of USP26 in patients with severely impaired spermatogenesis. Eur. J. Hum. Genet..

[22] Wright A., Reiley W.W., Chang M., Jin W., Lee A.J., Zhang M., Sun S.C. (2007). Regulation of early wave of germ cell apoptosis and spermatogenesis by deubiquitinating enzyme CYLD. Dev. Cell.

[23] Crimmins S., Sutovsky M., Chen P.C., Huffman A., Wheeler C., Swing D.A., Roth K., Wilson J., Sutovsky P., Wilson S. (2009). Transgenic rescue of ataxia mice reveals a male-specific sterility defect. Dev. Biol..

[24] Kim N., Xiao R., Choi H., Jo H., Kim J.H., Uhm S.J., Park C. (2011). Abnormal sperm development in pcd(3J)-/- mice: The importance of Agtpbp1 in spermatogenesis. Mol. Cells.

[25] Bedard N., Yang Y., Gregory M., Cyr D.G., Suzuki J., Yu X., Chian R.C., Hermo L., O’Flaherty C., Smith C.E. (2011). Mice lacking the USP2 deubiquitinating enzyme have severe male subfertility associated with defects in fertilization and sperm motility. Biol. Reprod..

[26] Manku G., Wing S.S., Culty M. (2012). Expression of the ubiquitin proteasome system in neonatal rat gonocytes and spermatogonia: Role in gonocyte differentiation. Biol. Reprod..

[27] Braun R.E. (2001). Packaging paternal chromosomes with protamine. Nat. Genet..

[28] Oliva R. (2006). Protamines and male infertility. Hum. Reprod. Update.

[29] Kierszenbaum A.L., Tres L.L. (2004). The acrosome-acroplaxome-manchette complex and the shaping of the spermatid head. Arch. Histol. Cytol..

[30] Ward W.S., Coffey D.S. (1991). DNA packaging and organization in mammalian spermatozoa: Comparison with somatic cells. Biol. Reprod..

[31] Baarends W.M., Hoogerbrugge J.W., Roest H.P., Ooms M., Vreeburg J., Hoeijmakers J.H., Grootegoed J.A. (1999). Histone ubiquitination and chromatin remodeling in mouse spermatogenesis. Dev. Biol..

[32] Sung P., Prakash S., Prakash L. (1988). The RAD6 protein of Saccharomyces cerevisiae polyubiquitinates histones, and its acidic domain mediates this activity. Genes Dev..

[33] Jentsch S., McGrath J.P., Varshavsky A. (1987). The yeast DNA repair gene RAD6 encodes a ubiquitin-conjugating enzyme. Nature.

[34] Robzyk K., Recht J., Osley M.A. (2000). Rad6-dependent ubiquitination of histone H2B in yeast. Science.

[35] Hwang W.W., Venkatasubrahmanyam S., Ianculescu A.G., Tong A., Boone C., Madhani H.D. (2003). A conserved RING finger protein required for histone H2B monoubiquitination and cell size control. Mol. Cell.

[36] Wood A., Krogan N.J., Dover J., Schneider J., Heidt J., Boateng M.A., Dean K., Golshani A., Zhang Y., Greenblatt J.F. (2003). Bre1, an E3 ubiquitin ligase required for recruitment and substrate selection of Rad6 at a promoter. Mol. Cell.

[37] Dover J., Schneider J., Tawiah-Boateng M.A., Wood A., Dean K., Johnston M., Shilatifard A. (2002). Methylation of histone H3 by COMPASS requires ubiquitination of histone H2B by Rad6. J. Biol. Chem..

[38] Sun Z.W., Allis C.D. (2002). Ubiquitination of histone H2B regulates H3 methylation and gene silencing in yeast. Nature.

[39] Briggs S.D., Xiao T., Sun Z.W., Caldwell J.A., Shabanowitz J., Hunt D.F., Allis C.D., Strahl B.D. (2002). Gene silencing: Trans-histone regulatory pathway in chromatin. Nature.

[40] Henry K.W., Wyce A., Lo W.S., Duggan L.J., Emre N.C., Kao C.F., Pillus L., Shilatifard A., Osley M.A., Berger S.L. (2003). Transcriptional activation via sequential histone H2B ubiquitylation and deubiquitylation, mediated by SAGA-associated Ubp8. Genes Dev..

[41] Kao C.F., Hillyer C., Tsukuda T., Henry K., Berger S., Osley M.A. (2004). Rad6 plays a role in transcriptional activation through ubiquitylation of histone H2B. Genes Dev..

[42] Wood A., Schneider J., Shilatifard A. (2005). Cross-talking histones: Implications for the regulation of gene expression and DNA repair. Biochem. Cell Biol..

[43] Pavri R., Zhu B., Li G., Trojer P., Mandal S., Shilatifard A., Reinberg D. (2006). Histone H2B monoubiquitination functions cooperatively with FACT to regulate elongation by RNA polymerase II. Cell.

[44] Osley M.A. (2006). Regulation of histone H2A and H2B ubiquitylation. Brief. Funct. Genomics Proteomics.

[45] Weake V.M., Workman J.L. (2008). Histone ubiquitination: Triggering gene activity. Mol. Cell.

[46] Koken M.H., Hoogerbrugge J.W., Jasper-Dekker I., de Wit J., Willemsen R., Roest H.P., Grootegoed J.A., Hoeijmakers J.H. (1996). Expression of the ubiquitin-conjugating DNA repair enzymes HHR6A and B suggests a role in spermatogenesis and chromatin modification. Dev. Biol..

[47] Roest H.P., van Klaveren J., de Wit J., van Gurp C.G., Koken M.H., Vermey M., van Roijen J.H., Hoogerbrugge J.W., Vreeburg J.T., Baarends W.M. (1996). Inactivation of the HR6B ubiquitin-conjugating DNA repair enzyme in mice causes male sterility associated with chromatin modification. Cell.

[48] Gaucher J., Reynoird N., Montellier E., Boussouar F., Rousseaux S., Khochbin S. (2010). From meiosis to postmeiotic events: The secrets of histone disappearance. FEBS J..

[49] Qian M.X., Pang Y., Liu C.H., Haratake K., Du B.Y., Ji D.Y., Wang G.F., Zhu Q.Q., Song W., Yu Y. (2013). Acetylation-mediated proteasomal degradation of core histones during DNA repair and spermatogenesis. Cell.

[50] Savulescu A.F., Glickman M.H. (2011). Proteasome activator 200: The heat is on. Mol. Cell. Proteomics.

[51] Khor B., Bredemeyer A.L., Huang C.Y., Turnbull I.R., Evans R., Maggi L.B., White J.M., Walker L.M., Carnes K., Hess R.A. (2006). Proteasome activator PA200 is required for normal spermatogenesis. Mol. Cell. Biol..

[52] Lu L.Y., Wu J., Ye L., Gavrilina G.B., Saunders T.L., Yu X. (2010). RNF8-dependent histone modifications regulate nucleosome removal during spermatogenesis. Dev. Cell.

[53] Baarends W.M., Wassenaar E., van der Laan R., Hoogerbrugge J., Sleddens-Linkels E., Hoeijmakers J.H., de Boer P., Grootegoed J.A. (2005). Silencing of unpaired chromatin and histone H2A ubiquitination in mammalian meiosis. Mol. Cell. Biol..

[54] Turner J.M., Mahadevaiah S.K., Fernandez-Capetillo O., Nussenzweig A., Xu X., Deng C.X., Burgoyne P.S. (2005). Silencing of unsynapsed meiotic chromosomes in the mouse. Nat. Genet..

[55] Kwon Y.T., Xia Z., An J.Y., Tasaki T., Davydov I.V., Seo J.W., Sheng J., Xie Y., Varshavsky A. (2003). Female lethality and apoptosis of spermatocytes in mice lacking the UBR2 ubiquitin ligase of the N-end rule pathway. Mol. Cell. Biol..

[56] An J.Y., Kim E.A., Jiang Y., Zakrzewska A., Kim D.E., Lee M.J., Mook-Jung I., Zhang Y., Kwon Y.T. (2010). UBR2 mediates transcriptional silencing during spermatogenesis via histone ubiquitination. Proc. Natl. Acad. Sci. USA.

[57] Mulugeta Achame E., Wassenaar E., Hoogerbrugge J.W., Sleddens-Linkels E., Ooms M., Sun Z.W., van I.W.F., Grootegoed J.A., Baarends W.M. (2010). The ubiquitin-conjugating enzyme HR6B is required for maintenance of X chromosome silencing in mouse spermatocytes and spermatids. BMC Genomics.

[58] Baarends W.M., Wassenaar E., Hoogerbrugge J.W., Schoenmakers S., Sun Z.W., Grootegoed J.A. (2007). Increased phosphorylation and dimethylation of XY body histones in the Hr6b-knockout mouse is associated with derepression of the X chromosome. J. Cell Sci..

[59] Baarends W.M., Wassenaar E., Hoogerbrugge J.W., van Cappellen G., Roest H.P., Vreeburg J., Ooms M., Hoeijmakers J.H., Grootegoed J.A. (2003). Loss of HR6B ubiquitin-conjugating activity results in damaged synaptonemal complex structure and increased crossing-over frequency during the male meiotic prophase. Mol. Cell. Biol..

[60] van der Laan R., Uringa E.J., Wassenaar E., Hoogerbrugge J.W., Sleddens E., Odijk H., Roest H.P., de Boer P., Hoeijmakers J.H., Grootegoed J.A. (2004). Ubiquitin ligase Rad18Sc localizes to the XY body and to other chromosomal regions that are unpaired and transcriptionally silenced during male meiotic prophase. J. Cell Sci..

[61] Kim J., Hake S.B., Roeder R.G. (2005). The human homolog of yeast BRE1 functions as a transcriptional coactivator through direct activator interactions. Mol. Cell.

[62] Liu Z., Oughtred R., Wing S.S. (2005). Characterization of E3Histone, a novel testis ubiquitin protein ligase which ubiquitinates histones. Mol. Cell. Biol..

[63] Liu Z., Miao D., Xia Q., Hermo L., Wing S.S. (2007). Regulated expression of the ubiquitin protein ligase, E3(Histone)/LASU1/Mule/ARF-BP1/HUWE1, during spermatogenesis. Dev. Dyn..

[64] Moreno R.D., Ramalho-Santos J., Chan E.K., Wessel G.M., Schatten G. (2000). The Golgi apparatus segregates from the lysosomal/acrosomal vesicle during rhesus spermiogenesis: Structural alterations. Dev. Biol..

[65] Ramalho-Santos J., Moreno R.D., Wessel G.M., Chan E.K., Schatten G. (2001). Membrane trafficking machinery components associated with the mammalian acrosome during spermiogenesis. Exp. Cell Res..

[66] Ramalho-Santos J., Moreno R.D. (2001). Targeting and fusion proteins during mammalian spermiogenesis. Biol. Res..

[67] Kang-Decker N., Mantchev G.T., Juneja S.C., McNiven M.A., van Deursen J.M. (2001). Lack of acrosome formation in Hrb-deficient mice. Science.

[68] Yao R., Ito C., Natsume Y., Sugitani Y., Yamanaka H., Kuretake S., Yanagida K., Sato A., Toshimori K., Noda T. (2002). Lack of acrosome formation in mice lacking a Golgi protein, GOPC. Proc. Natl. Acad. Sci. USA.

[69] Kierszenbaum A.L., Tres L.L., Rivkin E., Kang-Decker N., van Deursen J.M. (2004). The acroplaxome is the docking site of Golgi-derived myosin Va/Rab27a/b- containing proacrosomal vesicles in wild-type and Hrb mutant mouse spermatids. Biol. Reprod..

[70] Xiao N., Kam C., Shen C., Jin W., Wang J., Lee K.M., Jiang L., Xia J. (2009). PICK1 deficiency causes male infertility in mice by disrupting acrosome formation. J. Clin. Invest..

[71] Roqueta-Rivera M., Abbott T.L., Sivaguru M., Hess R.A., Nakamura M.T. (2011). Deficiency in the omega-3 fatty acid pathway results in failure of acrosome biogenesis in mice. Biol. Reprod..

[72] Lerer-Goldshtein T., Bel S., Shpungin S., Pery E., Motro B., Goldstein R.S., Bar-Sheshet S.I., Breitbart H., Nir U. (2010). TMF/ARA160: A key regulator of sperm development. Dev. Biol..

[73] Fridmann-Sirkis Y., Siniossoglou S., Pelham H.R. (2004). TMF is a golgin that binds Rab6 and influences Golgi morphology. BMC Cell Biol..

[74] Yamane J., Kubo A., Nakayama K., Yuba-Kubo A., Katsuno T., Tsukita S. (2007). Functional involvement of TMF/ARA160 in Rab6-dependent retrograde membrane traffic. Exp. Cell Res..

[75] Miller V.J., Sharma P., Kudlyk T.A., Frost L., Rofe A.P., Watson I.J., Duden R., Lowe M., Lupashin V.V., Ungar D. (2013). Molecular insights into vesicle tethering at the Golgi by the conserved oligomeric Golgi (COG) complex and the golgin TATA element modulatory factor (TMF). J. Biol. Chem..

[76] Perry E., Tsruya R., Levitsky P., Pomp O., Taller M., Weisberg S., Parris W., Kulkarni S., Malovani H., Pawson T. (2004). TMF/ARA160 is a BC-box-containing protein that mediates the degradation of Stat3. Oncogene.

[77] Abrham G., Volpe M., Shpungin S., Nir U. (2009). TMF/ARA160 downregulates proangiogenic genes and attenuates the progression of PC3 xenografts. Int. J. Cancer.

[78] Piper R.C., Katzmann D.J. (2007). Biogenesis and function of multivesicular bodies. Annu. Rev. Cell Dev. Biol..

[79] Haraguchi C.M., Mabuchi T., Hirata S., Shoda T., Hoshi K., Yokota S. (2004). Ubiquitin signals in the developing acrosome during spermatogenesis of rat testis: An immunoelectron microscopic study. J. Histochem. Cytochem..

[80] Morokuma Y., Nakamura N., Kato A., Notoya M., Yamamoto Y., Sakai Y., Fukuda H., Yamashina S., Hirata Y., Hirose S. (2007). MARCH-XI, a novel transmembrane ubiquitin ligase implicated in ubiquitin-dependent protein sorting in developing spermatids. J. Biol. Chem..

[81] Yogo K., Tojima H., Ohno J.Y., Ogawa T., Nakamura N., Hirose S., Takeya T., Kohsaka T. (2012). Identification of SAMT family proteins as substrates of MARCH11 in mouse spermatids. Histochem. Cell Biol..

[82] Nathan J.A., Lehner P.J. (2009). The trafficking and regulation of membrane receptors by the RING-CH ubiquitin E3 ligases. Exp. Cell Res..

[83] Tang X.M., Lalli M.F., Clermont Y. (1982). A cytochemical study of the Golgi apparatus of the spermatid during spermiogenesis in the rat. Am. J. Anat..

[84] Martinez-Menarguez J.A., Aviles M., Madrid J.F., Castells M.T., Ballesta J. (1993). Glycosylation in Golgi apparatus of early spermatids of rat. A high resolution lectin cytochemical study. Eur. J. Cell Biol..

[85] Iyengar P.V., Hirota T., Hirose S., Nakamura N. (2011). Membrane-associated RING-CH 10 (MARCH10 protein) is a microtubule-associated E3 ubiquitin ligase of the spermatid flagella. J. Biol. Chem..

[86] Zhao B., Ito K., Iyengar P.V., Hirose S., Nakamura N. (2013). MARCH7 E3 ubiquitin ligase is highly expressed in developing spermatids of rats and its possible involvement in head and tail formation. Histochem. Cell Biol..

[87] West A.P., Willison K.R. (1996). Brefeldin A and mannose 6-phosphate regulation of acrosomic related vesicular trafficking. Eur. J. Cell Biol..

[88] Li Y.C., Hu X.Q., Zhang K.Y., Guo J., Hu Z.Y., Tao S.X., Xiao L.J., Wang Q.Z., Han C.S., Liu Y.X. (2006). Afaf, a novel vesicle membrane protein, is related to acrosome formation in murine testis. FEBS Lett..

[89] Li S., Qiao Y., Di Q., Le X., Zhang L., Zhang X., Zhang C., Cheng J., Zong S., Koide S.S. (2009). Interaction of SH3P13 and DYDC1 protein: A germ cell component that regulates acrosome biogenesis during spermiogenesis. Eur. J. Cell Biol..

[90] Berruti G., Ripolone M., Ceriani M. (2010). USP8, a regulator of endosomal sorting, is involved in mouse acrosome biogenesis through interaction with the spermatid ESCRT-0 complex and microtubules. Biol. Reprod..

[91] Berruti G., Martegani E. (2005). The deubiquitinating enzyme mUBPy interacts with the sperm-specific molecular chaperone MSJ-1: The relation with the proteasome, acrosome, and centrosome in mouse male germ cells. Biol. Reprod..

[92] Wright M.H., Berlin I., Nash P.D. (2011). Regulation of endocytic sorting by ESCRT-DUB-mediated deubiquitination. Cell Biochem. Biophys..

[93] Bonifacino J.S., Hierro A. (2011). Transport according to GARP: Receiving retrograde cargo at the trans-Golgi network. Trends Cell Biol..

[94] Heimann P., Laage S., Jockusch H. (1991). Defect of sperm assembly in a neurological mutant of the mouse, wobbler (WR). Differentiation.

[95] Leestma J.E., Sepsenwol S. (1980). Sperm tail axoneme alterations in the Wobbler mouse. J. Reprod. Fertil..

[96] Schmitt-John T., Drepper C., Mussmann A., Hahn P., Kuhlmann M., Thiel C., Hafner M., Lengeling A., Heimann P., Jones J.M. (2005). Mutation of Vps54 causes motor neuron disease and defective spermiogenesis in the wobbler mouse. Nat. Genet..

[97] Paiardi C., Pasini M.E., Gioria M., Berruti G. (2011). Failure of acrosome formation and globozoospermia in the wobbler mouse, a Vps54 spontaneous recessive mutant. Spermatogenesis.

[98] Kierszenbaum A.L., Rivkin E., Tres L.L. (2003). Acroplaxome, an F-actin-keratin-containing plate, anchors the acrosome to the nucleus during shaping of the spermatid head. Mol. Biol. Cell.

[99] Yang W.X., Jefferson H., Sperry A.O. (2006). The molecular motor KIFC1 associates with a complex containing nucleoporin NUP62 that is regulated during development and by the small GTPase RAN. Biol. Reprod..

[100] Saade M., Irla M., Govin J., Victorero G., Samson M., Nguyen C. (2007). Dynamic distribution of Spatial during mouse spermatogenesis and its interaction with the kinesin KIF17b. Exp. Cell Res..

[101] Lehti M.S., Kotaja N., Sironen A. (2013). KIF3A is essential for sperm tail formation and manchette function. Mol. Cell. Endocrinol..

[102] Hall E.S., Eveleth J., Jiang C., Redenbach D.M., Boekelheide K. (1992). Distribution of the microtubule-dependent motors cytoplasmic dynein and kinesin in rat testis. Biol. Reprod..

[103] Yoshida T., Ioshii S.O., Imanaka-Yoshida K., Izutsu K. (1994). Association of cytoplasmic dynein with manchette microtubules and spermatid nuclear envelope during spermiogenesis in rats. J. Cell. Sci..

[104] Rivkin E., Kierszenbaum A.L., Gil M., Tres L.L. (2009). Rnf19a, a ubiquitin protein ligase, and Psmc3, a component of the 26S proteasome, tether to the acrosome membranes and the head-tail coupling apparatus during rat spermatid development. Dev. Dyn..

[105] Federico A., Cardaioli E., Da Pozzo P., Formichi P., Gallus G.N., Radi E. (2012). Mitochondria, oxidative stress and neurodegeneration. J. Neurol. Sci..

[106] Ramalho-Santos J., Varum S., Amaral S., Mota P.C., Sousa A.P., Amaral A. (2009). Mitochondrial functionality in reproduction: From gonads and gametes to embryos and embryonic stem cells. Hum. Reprod. Update.

[107] Piomboni P., Focarelli R., Stendardi A., Ferramosca A., Zara V. (2012). The role of mitochondria in energy production for human sperm motility. Int. J. Androl..

[108] Amaral A., Lourenco B., Marques M., Ramalho-Santos J. (2013). Mitochondria functionality and sperm quality. Reproduction.

[109] Gyllensten U., Wharton D., Josefsson A., Wilson A.C. (1991). Paternal inheritance of mitochondrial DNA in mice. Nature.

[110] Kaneda H., Hayashi J., Takahama S., Taya C., Lindahl K.F., Yonekawa H. (1995). Elimination of paternal mitochondrial DNA in intraspecific crosses during early mouse embryogenesis. Proc. Natl. Acad. Sci. USA.

[111] Shitara H., Hayashi J.I., Takahama S., Kaneda H., Yonekawa H. (1998). Maternal inheritance of mouse mtDNA in interspecific hybrids: Segregation of the leaked paternal mtDNA followed by the prevention of subsequent paternal leakage. Genetics.

[112] Piko L., Taylor K.D. (1987). Amounts of mitochondrial DNA and abundance of some mitochondrial gene transcripts in early mouse embryos. Dev. Biol..

[113] Hecht N.B., Liem H., Kleene K.C., Distel R.J., Ho S.M. (1984). Maternal inheritance of the mouse mitochondrial genome is not mediated by a loss or gross alteration of the paternal mitochondrial DNA or by methylation of the oocyte mitochondrial DNA. Dev. Biol..

[114] Shitara H., Kaneda H., Sato A., Inoue K., Ogura A., Yonekawa H., Hayashi J.I. (2000). Selective and continuous elimination of mitochondria microinjected into mouse eggs from spermatids, but not from liver cells, occurs throughout embryogenesis. Genetics.

[115] Sutovsky P., Moreno R.D., Ramalho-Santos J., Dominko T., Simerly C., Schatten G. (1999). Ubiquitin tag for sperm mitochondria. Nature.

[116] Sutovsky P., Moreno R.D., Ramalho-Santos J., Dominko T., Simerly C., Schatten G. (2000). Ubiquitinated sperm mitochondria, selective proteolysis, and the regulation of mitochondrial inheritance in mammalian embryos. Biol. Reprod..

[117] Luo S.M., Ge Z.J., Wang Z.W., Jiang Z.Z., Wang Z.B., Ouyang Y.C., Hou Y., Schatten H., Sun Q.Y. (2013). Unique insights into maternal mitochondrial inheritance in mice. Proc. Natl. Acad. Sci. USA.

[118] Yonashiro R., Ishido S., Kyo S., Fukuda T., Goto E., Matsuki Y., Ohmura-Hoshino M., Sada K., Hotta H., Yamamura H. (2006). A novel mitochondrial ubiquitin ligase plays a critical role in mitochondrial dynamics. EMBO J..

[119] Nakamura N., Kimura Y., Tokuda M., Honda S., Hirose S. (2006). MARCH-V is a novel mitofusin 2- and Drp1-binding protein able to change mitochondrial morphology. EMBO Rep..

[120] Ashrafi G., Schwarz T.L. (2013). The pathways of mitophagy for quality control and clearance of mitochondria. Cell Death Differ..

[121] Feng D., Liu L., Zhu Y., Chen Q. (2013). Molecular signaling toward mitophagy and its physiological significance. Exp. Cell Res..

[122] Sato M., Sato K. (2011). Degradation of paternal mitochondria by fertilization-triggered autophagy in *C. elegans* embryos. Science.

[123] Al Rawi S., Louvet-Vallee S., Djeddi A., Sachse M., Culetto E., Hajjar C., Boyd L., Legouis R., Galy V. (2011). Postfertilization autophagy of sperm organelles prevents paternal mitochondrial DNA transmission. Science.

[124] Tsukamoto S., Kuma A., Murakami M., Kishi C., Yamamoto A., Mizushima N. (2008). Autophagy is essential for preimplantation development of mouse embryos. Science.

[125] Okatsu K., Saisho K., Shimanuki M., Nakada K., Shitara H., Sou Y.S., Kimura M., Sato S., Hattori N., Komatsu M. (2010). p62/SQSTM1 cooperates with Parkin for perinuclear clustering of depolarized mitochondria. Genes Cells.

[126] Narendra D., Kane L.A., Hauser D.N., Fearnley I.M., Youle R.J. (2010). p62/SQSTM1 is required for Parkin-induced mitochondrial clustering but not mitophagy; VDAC1 is dispensable for both. Autophagy.

[127] Escalier D. (2003). New insights into the assembly of the periaxonemal structures in mammalian spermatozoa. Biol. Reprod..

[128] Hermo L., Pelletier R.M., Cyr D.G., Smith C.E. (2010). Surfing the wave, cycle, life history, and genes/proteins expressed by testicular germ cells. Part 4: Intercellular bridges, mitochondria, nuclear envelope, apoptosis, ubiquitination, membrane/voltage-gated channels, methylation/acetylation, and transcription factors. Microsc. Res. Tech..

[129] Sutovsky P. (2003). Ubiquitin-dependent proteolysis in mammalian spermatogenesis, fertilization, and sperm quality control: Killing three birds with one stone. Microsc. Res. Tech..

[130] Sutovsky P. (2011). Sperm proteasome and fertilization. Reproduction.

